# Supplementary material for: Pitch characteristics of real-world infant-directed speech vary with pragmatic context, perceived adult gender, and infant gender
Source: PLoS One. 2025 Jun 25;20(6):e0326569. doi: 10.1371/journal.pone.0326569 (PMC12192138; doi:10.1371/journal.pone.0326569)
Supplement: S1 File — Supplementary Material. (DOCX) [file pone.0326569.s001.docx]

**Supplemental Material**

**Pragmatic Categories Descriptive Data**

| Pragmatic Context | | | |
| --- | --- | --- | --- |
|  | Number of Clips |  | Number of Clips Marked as Context & Noisy* |
| Inform | 2040 |  | 25 |
| Conversational Basics | 685 |  | 32 |
| Question | 745 |  | 18 |
| Imperative | 392 |  | 8 |
| Reading | 232 |  | 0 |
| Singing | 226 |  | 0 |
| Comfort | 42 |  | 1 |
| Vocal Play | 37 |  | 1 |

**Note.* Clips were excluded if they were exclusively coded as noisy and did not carry a context code. Here we have denoted the number of clips per pragmatic context and of those clips how many also carried a noisy code. This often occurred when part of the clip was decipherable, and part of the clip was indecipherable.

**Regression Tables**

The following regression output tables were generated using *sjPlot* (2.8.14; Lüdecke, 2023) in R. Interaction plots visualize post-hoc pairwise comparisons. Mean pitch and standard deviation of pitch (i.e., pitch variability), log-transformed, group-mean centered, and scaled by one standard deviation, are plotted on the y-axis. Interaction plots were created using the *ggeffects* package (1.3.0.4; Lüdecke, 2018).

Lüdecke, D. (2023). sjPlot: Data Visualization for Statistics in Social Science. R package version 2.8.14, [https://CRAN.R-project.org/package=sjPlot](https://cran.r-project.org/package=sjPlot).

Lüdecke, D. (2018). ggeffects: Tidy Data Frames of Marginal Effects from Regression Models. Journal of Open Source Software, 3(26), 772. doi: [10.21105/joss.00772](https://doi.org/10.21105/joss.00772)

**Prevalence of Pragmatic Contexts Logistic Mixed Effects Model Output**

|  | **Proportion** | | | |
| --- | --- | --- | --- | --- |
| *Predictors* | *Odds Ratios* | *SE* | *CI* | *p* |
| (Intercept) | 10.20 | 1.70 | 0.01 – Inf | **.701** |
| Adult Gender | 1.45 | 0.25 | 0.00 – Inf | .950 |
| Conversational Basics | 0.88 | 0.04 | 0.80 – 0.96 | **.007** |
| Comfort | 1.69 | 55.37 | 0.00 – Inf | .972 |
| Sing | 2.52 | 0.55 | 1.88 – 4.11 | **< .001** |
| Inform | 0.58 | 0.04 | 0.51 – 0.65 | **< .001** |
| Imperative | 1.73 | 0.11 | 1.52 – 1.96 | **< .001** |
| Question | 1.25 | 0.07 | 1.12 – 1.39 | **< .001** |
| Reading | 19.40 | 1435.30 | 0.00 – Inf | .895 |
| Vocal Play | 2.52 | 118.86 | 0.00 – Inf | **.965** |
| Gender*Conversational Basics | 1.05 | 0.05 | 0.96 – 1.14 | .334 |
| Gender*Comfort | 2.08 | 116.12 | 0.00 – Inf | .977 |
| Gender*Sing | 0.95 | 0.15 | 0.70 – 1.30 | .757 |
| Gender*Inform | 0.97 | 0.06 | 0.86 – 1.09 | .615 |
| Gender*Imperative | 0.97 | 0.07 | 0.85 – 1.11 | .680 |
| Gender*Question | 0.90 | 0.05 | 0.811 – 9.99 | **.050** |
| Gender*Reading | 2.70 | 155.52 | 0.00 – Inf | .945 |
| Gender*Vocal Play | 1.50 | 102.62 | 0.00 – Inf | .975 |
| **Random Effects** | | | | |
| τ_00_ _ID_ | 1.95 | | | |
| τ_00_ _coder_ | 0.06 | | | |
| N _coder_ | 4 | | | |
| N _ID_ | 60 | | | |
| Observations | 3607 | | | |

***Perceived Adult Speaker Gender and Register Analysis Within Specific Pragmatic Contexts***

**Inform Context**

|  | **Mean Pitch** | | | |
| --- | --- | --- | --- | --- |
| *Predictors* | *Estimates* | *SE* | *CI* | *p* |
| (Intercept) | 0.01 | 0.05 | -0.10 – 0.11 | .905 |
| Adult Gender | -0.43 | 0.02 | -0.47 – -0.38 | **< .001** |
| Register | 0.19 | 0.02 | 0.15 – 0.23 | **< .001** |
| Adult Gender*Register | -0.09 | 0.02 | -0.13 – -0.05 | **< .001** |
| **Random Effects** | | | | |
| σ^2^ | 0.62 | | | |
| τ_00_ _ID_ | 0.11 | | | |
| τ_00_ _coder_ | 0.00 | | | |
| ICC | 0.15 | | | |
| N _ID_ | 60 | | | |
| N _coder_ | 4 | | | |
| Observations | 2040 | | | |
| Marginal R^2^ / Conditional R^2^ | 0.207 / 0.330 | | | |

| **Interaction Contrasts** | **Levels** | **Estimate** | **SE** | **DF** | **lower.CL** | **upper.CL** | **t.ratio** | **p.value** |
| --- | --- | --- | --- | --- | --- | --- | --- | --- |
| Female-Male | ADS | 0.72 | 0.07 | 1989.87 | 0.57 | 0.86 | 11.07 | < .001 |
| Female-Male | IDS | 1.13 | 0.08 | 1396.37 | 0.96 | 1.30 | 14.53 | < .001 |
| IDS-ADS | Female | 0.49 | 0.05 | 1764.29 | 0.38 | 0.61 | 9.52 | < .001 |
| IDS-ADS | Male | 0.08 | 0.08 | 1775.94 | -0.11 | 0.26 | 0.92 | 0.36 |


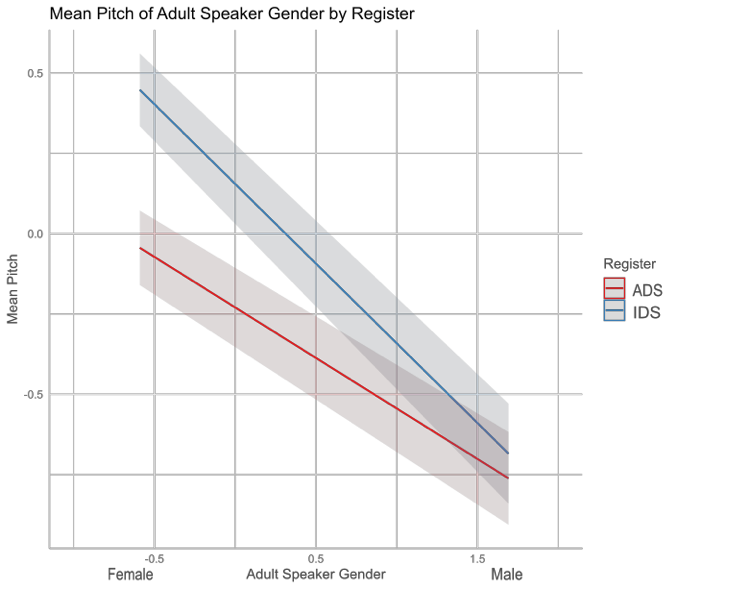

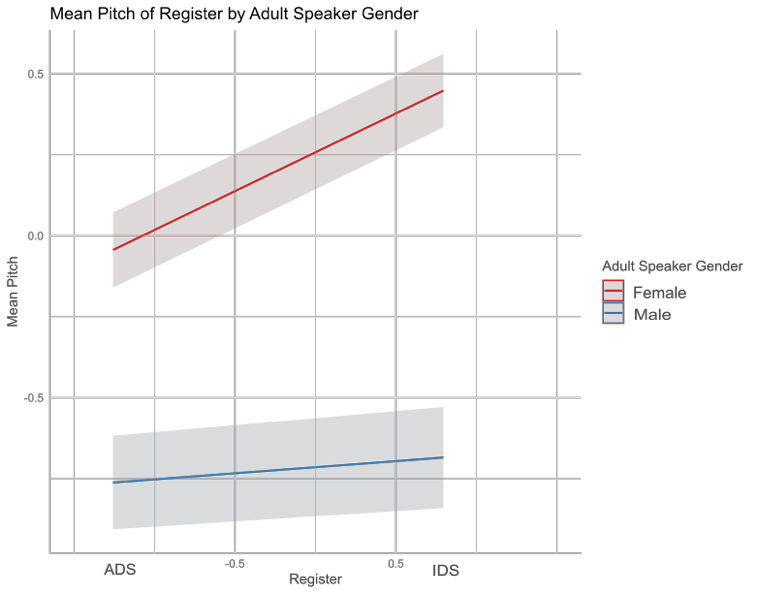


|  | **Pitch Variability** | | | |
| --- | --- | --- | --- | --- |
| *Predictors* | *Estimates* | *SE* | *CI* | *p* |
| (Intercept) | 0.02 | 0.06 | -0.11 – 0.16 | .681 |
| Adult Gender | -0.04 | 0.03 | -0.10 – 0.01 | .104 |
| Register | 0.14 | 0.02 | 0.09 – 0.19 | **< .001** |
| Adult Gender*Register | -0.04 | 0.02 | -0.08 – 0.01 | .128 |
| **Random Effects** | | | | |
| σ^2^ | 0.87 | | | |
| τ_00_ _ID_ | 0.06 | | | |
| τ_00_ _coder_ | 0.00 | | | |
| ICC | 0.07 | | | |
| N _ID_ | 60 | | | |
| N _coder_ | 4 | | | |
| Observations | 2040 | | | |
| Marginal R^2^ / Conditional R^2^ | 0.023 / 0.092 | | | |

**Conversational Basics Context**

|  | **Mean Pitch** | | | |
| --- | --- | --- | --- | --- |
| *Predictors* | *Estimates* | *SE* | *CI* | *p* |
| (Intercept) | 0.10 | 0.08 | -0.06 – 0.27 | .206 |
| Adult Gender | -0.33 | 0.04 | -0.40 – -0.25 | **< .001** |
| Register | 0.16 | 0.04 | 0.09 – 0.23 | **< .001** |
| Adult Gender*Register | -0.11 | 0.03 | -0.18 – -0.04 | **.001** |
| **Random Effects** | | | | |
| σ^2^ | 0.63 | | | |
| τ_00_ _ID_ | 0.15 | | | |
| τ_00_ _coder_ | 0.01 | | | |
| ICC | 0.20 | | | |
| N _ID_ | 57 | | | |
| N _coder_ | 4 | | | |
| Observations | 685 | | | |
| Marginal R^2^ / Conditional R^2^ | 0.144 / 0.317 | | | |

| **Interaction Contrasts** | **Levels** | **Estimate** | **SE** | **DF** | **lower.CL** | **upper.CL** | **t.ratio** | **p.value** |
| --- | --- | --- | --- | --- | --- | --- | --- | --- |
| Female-Male | ADS | 0.43 | 0.12 | 680 | 0.16 | 0.71 | 3.52 | < .001 |
| Female-Male | IDS | 0.95 | 0.12 | 503 | 0.68 | 1.23 | 8.05 | < .001 |
| IDS-ADS | Female | 0.45 | 0.09 | 632 | 0.26 | 0.65 | 5.21 | < .001 |
| IDS-ADS | Male | -0.06 | 0.14 | 676 | -0.37 | 0.25 | -0.42 | 0.68 |


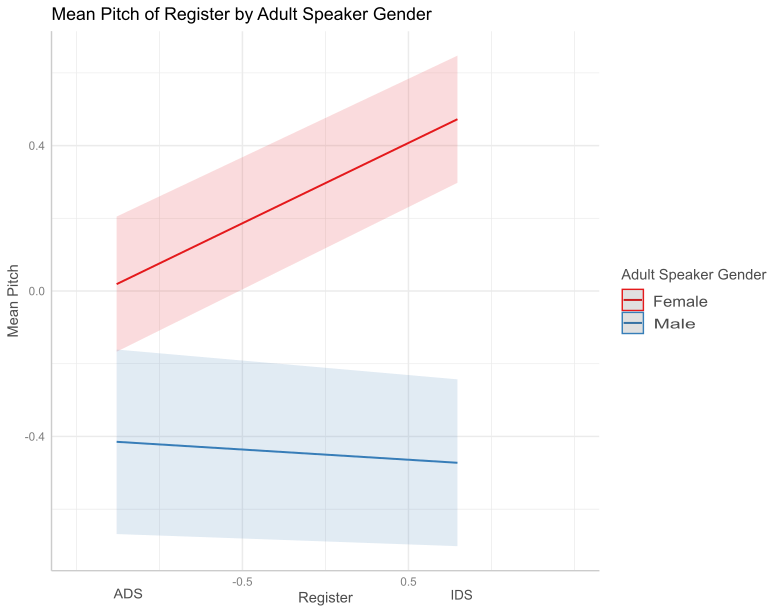

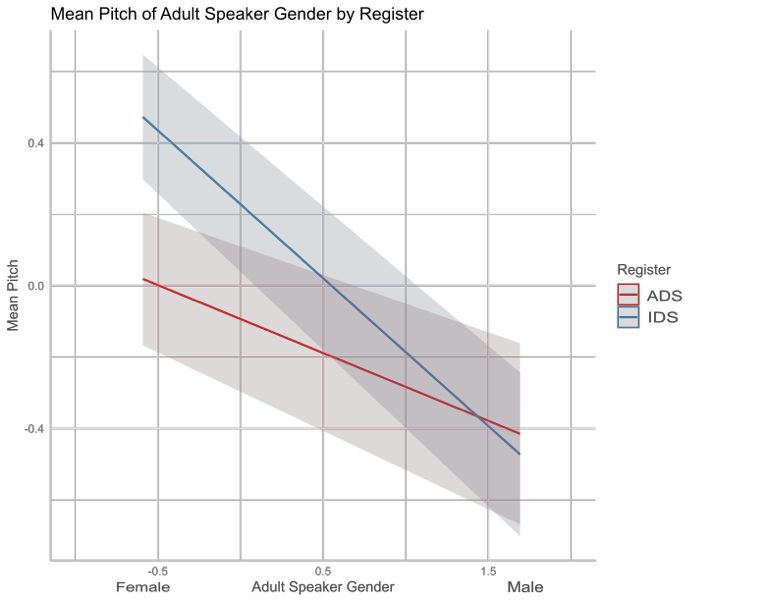


|  | **Pitch Variability** | | | |
| --- | --- | --- | --- | --- |
| *Predictors* | *Estimates* | *SE* | *CI* | *p* |
| (Intercept) | 0.11 | 0.09 | -0.12 – 0.34 | .265 |
| Adult Gender | -0.06 | 0.04 | -0.13 – 0.02 | .149 |
| Register | 0.08 | 0.04 | 0.01 – 0.16 | **.020** |
| Adult Gender*Register | -0.03 | 0.03 | -0.10 – 0.04 | .391 |
| **Random Effects** | | | | |
| σ^2^ | 0.69 | | | |
| τ_00_ _ID_ | 0.08 | | | |
| τ_00_ _coder_ | 0.02 | | | |
| ICC | 0.12 | | | |
| N _ID_ | 57 | | | |
| N _coder_ | 4 | | | |
| Observations | 685 | | | |
| Marginal R^2^ / Conditional R^2^ | 0.013 / 0.134 | | | |

**Question Context**

|  | **Mean Pitch** | | | |
| --- | --- | --- | --- | --- |
| *Predictors* | *Estimates* | *SE* | *CI* | *p* |
| (Intercept) | 0.02 | 0.06 | -0.09 – 0.14 | .665 |
| Adult Gender | -0.48 | 0.04 | -0.55 – -0.41 | **< .001** |
| Register | 0.19 | 0.04 | 0.12 – 0.26 | **< .001** |
| Adult Gender*Register | -0.02 | 0.03 | -0.09 – 0.05 | .565 |
| **Random Effects** | | | | |
| σ^2^ | 0.65 | | | |
| τ_00_ _ID_ | 0.11 | | | |
| τ_00_ _coder_ | 0.00 | | | |
| N _ID_ | 60 | | | |
| N _coder_ | 4 | | | |
| Observations | 745 | | | |
| Marginal R^2^ / Conditional R^2^ | 0.289 / NA | | | |

|  | **Pitch Variability** | | | |
| --- | --- | --- | --- | --- |
| *Predictors* | *Estimates* | *SE* | *CI* | *p* |
| (Intercept) | 0.07 | 0.05 | -0.03 – 0.18 | .170 |
| Adult Gender | -0.11 | 0.04 | -0.18 – -0.03 | **.006** |
| Register | 0.06 | 0.04 | -0.02 – 0.14 | .122 |
| Adult Gender*Register | 0.03 | 0.04 | -0.04 – 0.11 | .358 |
| **Random Effects** | | | | |
| σ^2^ | 0.78 | | | |
| τ_00_ _ID_ | 0.08 | | | |
| τ_00_ _coder_ | 0.00 | | | |
| N _ID_ | 60 | | | |
| N _coder_ | 4 | | | |
| Observations | 745 | | | |
| Marginal R^2^ / Conditional R^2^ | 0.018 / NA | | | |

**Imperative Context**

|  | **Mean Pitch** | | | |
| --- | --- | --- | --- | --- |
| *Predictors* | *Estimates* | *SE* | *CI* | *p* |
| (Intercept) | 0.14 | 0.09 | -0.04 – 0.33 | .117 |
| Adult Gender | -0.43 | 0.07 | -0.57 – -0.30 | **< .001** |
| Register | 0.07 | 0.07 | -0.06 – 0.20 | .311 |
| Adult Gender*Register | -0.04 | 0.07 | -0.17 – 0.09 | .538 |
| **Random Effects** | | | | |
| σ^2^ | 0.46 | | | |
| τ_00_ _ID_ | 0.24 | | | |
| τ_00_ _coder_ | 0.00 | | | |
| N _ID_ | 51 | | | |
| N _coder_ | 4 | | | |
| Observations | 392 | | | |
| Marginal R^2^ / Conditional R^2^ | 0.293 / NA | | | |
|  |  | | | |

|  | **Pitch Variability** | | | |
| --- | --- | --- | --- | --- |
| *Predictors* | *Estimates* | *SE* | *CI* | *p* |
| (Intercept) | 0.07 | 0.08 | -0.09 – 0.24 | .372 |
| Adult Gender | 0.07 | 0.08 | -0.08 – 0.22 | .377 |
| Register | 0.11 | 0.08 | -0.04 – 0.27 | .160 |
| Adult Gender*Register | -0.15 | 0.08 | -0.31 – 0.01 | .067 |
| **Random Effects** | | | | |
| σ^2^ | 0.77 | | | |
| τ_00_ _ID_ | 0.10 | | | |
| τ_00_ _coder_ | 0.00 | | | |
| N _ID_ | 51 | | | |
| N _coder_ | 4 | | | |
| Observations | 392 | | | |
| Marginal R^2^ / Conditional R^2^ | 0.020 / NA | | | |

***Comparison of Perceived Adult Speaker Gender and Pragmatic Context within IDS***

|  | **Mean Pitch** | | | |
| --- | --- | --- | --- | --- |
| *Predictors* | *Estimates* | *SE* | *CI* | *p* |
| (Intercept) | 0.20 | 0.07 | 0.03 – 0.36 | **.025** |
| Adult Gender | -0.47 | 0.03 | -0.52 – -0.42 | **< .001** |
| Conversational Basics | 0.03 | 0.02 | -0.01 – 0.07 | .092 |
| Singing | -0.01 | 0.02 | -0.05 – 0.03 | .535 |
| Inform | -0.01 | 0.02 | -0.06 – 0.03 | .566 |
| Reading | -0.04 | 0.02 | -0.07 – 0.00 | .068 |
| Imperative | 0.01 | 0.02 | -0.02 – 0.05 | .496 |
| Questions | 0.00 | 0.02 | -0.04 – 0.04 | .956 |
| Comfort | 0.02 | 0.02 | -0.02 – 0.05 | .276 |
| Adult Gender*Conversational Basics | 0.02 | 0.02 | -0.02 – 0.05 | .431 |
| Adult Gender*Singing | -0.05 | 0.02 | -0.08 – -0.01 | **.007** |
| Adult Gender*Inform | -0.03 | 0.02 | -0.07 – 0.02 | .203 |
| Adult Gender*Reading | -0.03 | 0.02 | -0.06 – 0.01 | .136 |
| Adult Gender*Imperative | 0.00 | 0.02 | -0.03 – 0.04 | .795 |
| Adult Gender*Questions | -0.02 | 0.02 | -0.06 – 0.02 | .244 |
| Adult Gender*Comfort | -0.03 | 0.01 | -0.06 – 0.00 | .054 |
| **Random Effects** | | | | |
| σ^2^ | 0.68 | | | |
| τ_00_ _ID_ | 0.09 | | | |
| τ_00_ _coder_ | 0.01 | | | |
| ICC | 0.13 | | | |
| N _ID_ | 60 | | | |
| N _coder_ | 4 | | | |
| Observations | 2210 | | | |
| Marginal R^2^ / Conditional R^2^ | 0.243 / 0.344 | | | |

**Perceived Adult Speaker Gender*Singing Context Interaction**

| **Interaction Contrasts** | **Levels** | **Estimate** | **SE** | **DF** | **lower.CL** | **upper.CL** | **t.ratio** | **p.value** |
| --- | --- | --- | --- | --- | --- | --- | --- | --- |
| Female-Male | Non-Singing | 1.44 | 0.21 | 2122 | 0.97 | 1.91 | 6.88 | <.001 |
| Female-Male | Singing | 1.90 | 0.28 | 2176 | 1.27 | 2.52 | 6.79 | < .001 |
| Singing-Non-Singing | Female | 0.07 | 0.09 | 2143 | -0.14 | 0.28 | 0.734 | .46 |
| Singing-Non-Singing | Male | -0.39 | 0.14 | 2099 | -0.71 | -0.07 | -2.72 | .01 |


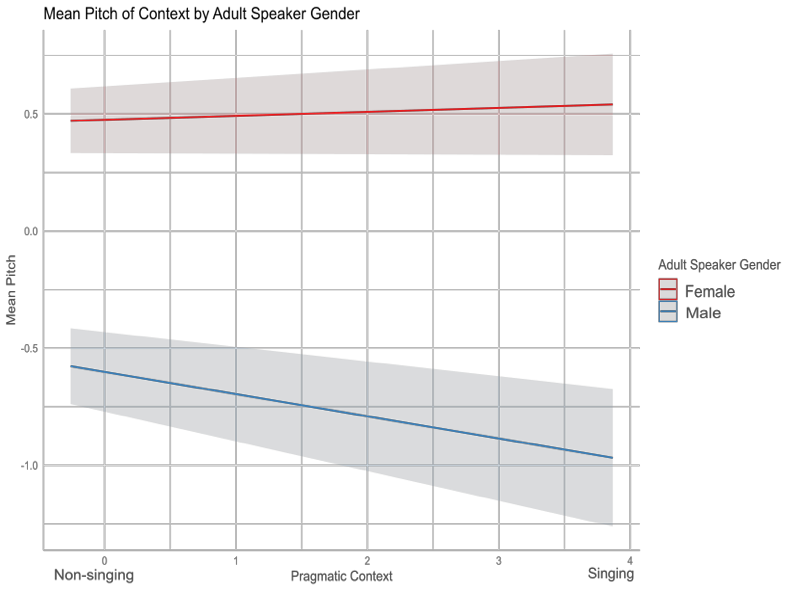

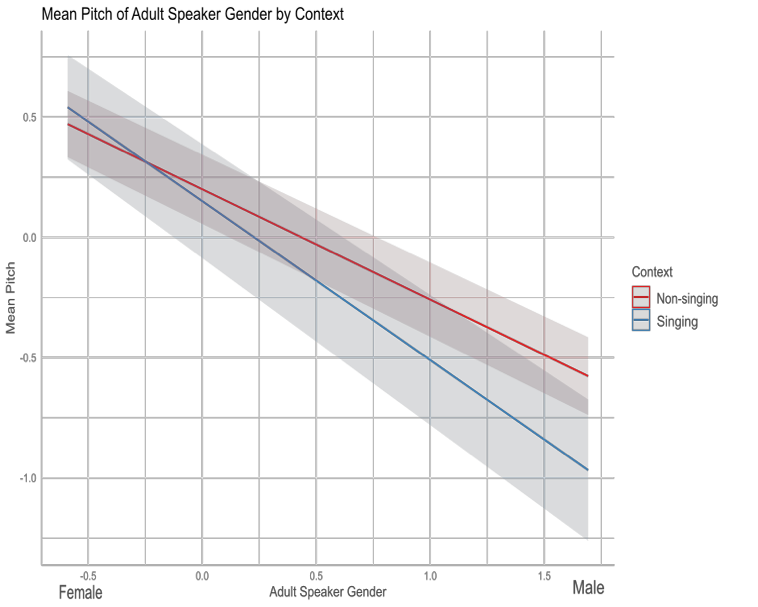


|  | **Pitch Variability** | | | |
| --- | --- | --- | --- | --- |
| *Predictors* | *Estimates* | *SE* | *CI* | *p* |
| (Intercept) | 0.11 | 0.04 | 0.04 – 0.18 | **.004** |
| Adult Gender | -0.09 | 0.03 | -0.14 – -0.04 | **.001** |
| Conversational Basics | 0.05 | 0.02 | 0.00 – 0.09 | **.032** |
| Singing | -0.19 | 0.02 | -0.23 – -0.15 | **< .001** |
| Inform | -0.02 | 0.03 | -0.07 – 0.03 | .384 |
| Reading | 0.01 | 0.02 | -0.03 – 0.05 | .768 |
| Imperative | 0.00 | 0.02 | -0.03 – 0.04 | .833 |
| Questions | -0.00 | 0.02 | -0.05 – 0.04 | .932 |
| Comfort | 0.06 | 0.02 | 0.02 – 0.09 | **.004** |
| Adult Gender*Conversational Basics | 0.01 | 0.02 | -0.03 – 0.05 | .674 |
| Adult Gender*Singing | -0.06 | 0.02 | -0.09 – -0.02 | **.005** |
| Adult Gender*Inform | 0.02 | 0.02 | -0.03 – 0.07 | .406 |
| Adult Gender*Reading | -0.01 | 0.02 | -0.05 – 0.03 | .629 |
| Adult Gender*Imperative | 0.01 | 0.02 | -0.02 – 0.05 | .466 |
| Adult Gender*Questions | 0.01 | 0.02 | -0.03 – 0.06 | .509 |
| Adult Gender*Comfort | -0.01 | 0.02 | -0.04 – 0.02 | .572 |
| **Random Effects** | | | | |
| σ^2^ | 0.84 | | | |
| τ_00_ _ID_ | 0.05 | | | |
| τ_00_ _coder_ | 0.00 | | | |
| N _ID_ | 60 | | | |
| N _coder_ | 4 | | | |
| Observations | 2210 | | | |
| Marginal R^2^ / Conditional R^2^ | 0.087 / NA | | | |

**Perceived Adult Speaker Gender*Singing Context Interaction**

| **Interaction Contrasts** | **Levels** | **Estimate** | **SE** | **DF** | **lower.CL** | **upper.CL** | **t.ratio** | **p.value** |
| --- | --- | --- | --- | --- | --- | --- | --- | --- |
| Female-Male | Non-Singing | 0.23 | 0.23 | 1784 | -0.28 | 0.74 | 0.99 | .32 |
| Female-Male | Singing | 0.75 | 0.31 | 1558 | 0.05 | 1.44 | 2.42 | .03 |
| Singing-Non-Singing | Female | -0.65 | 0.10 | 1524 | -0.88 | -0.42 | -6.27 | <.001 |
| Singing-Non-Singing | Male | -1.17 | 0.16 | 1836 | -1.52 | -0.82 | -7.47 | <.001 |

***
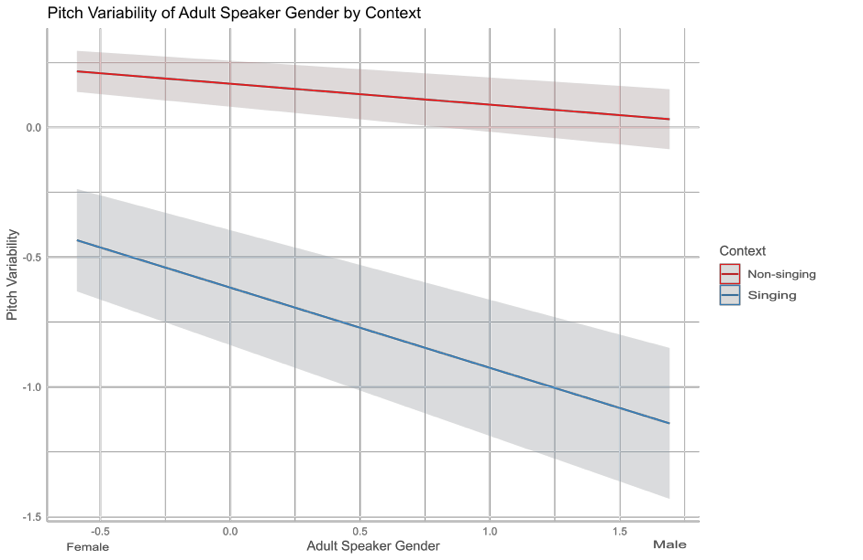

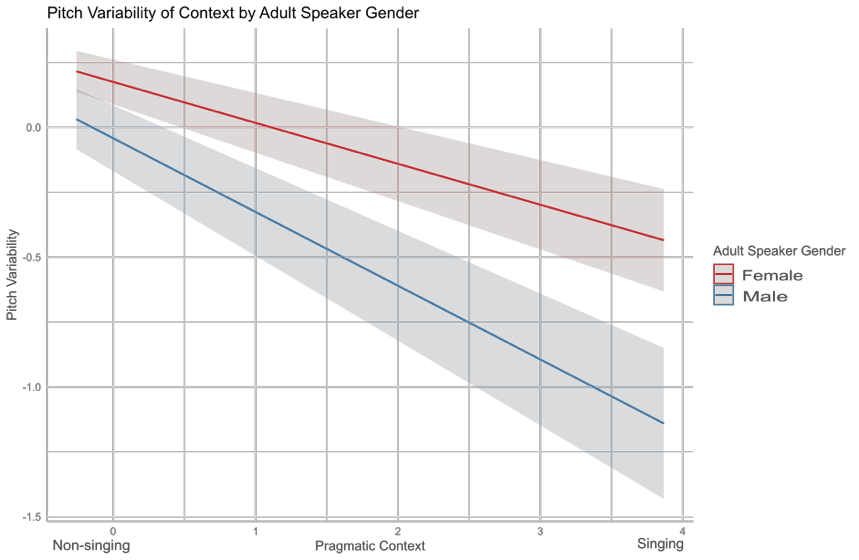
***

***Perceived Adult Speaker Gender and Assigned Infant Gender in IDS***

**Inform Context**

|  | **Mean Pitch** | | | |
| --- | --- | --- | --- | --- |
| *Predictors* | *Estimates* | *SE* | *CI* | *p* |
| (Intercept) | 0.17 | 0.06 | 0.04 – 0.30 | **.018** |
| Adult Gender | -0.52 | 0.04 | -0.59 – -0.44 | **< .001** |
| Infant Gender | 0.03 | 0.05 | -0.06 – 0.13 | .482 |
| Adult Gender*Infant Gender | 0.02 | 0.04 | -0.05 – 0.10 | .513 |
| **Random Effects** | | | | |
| σ^2^ | 0.64 | | | |
| τ_00_ _ID_ | 0.08 | | | |
| τ_00_ _coder_ | 0.00 | | | |
| ICC | 0.12 | | | |
| N _ID_ | 59 | | | |
| N _coder_ | 4 | | | |
| Observations | 900 | | | |
| Marginal R^2^ / Conditional R^2^ | 0.276 / 0.362 | | | |

|  | **Pitch Variability** | | | |
| --- | --- | --- | --- | --- |
| *Predictors* | *Estimates* | *SE* | *CI* | *p* |
| (Intercept) | 0.15 | 0.05 | 0.05 – 0.25 | **.006** |
| Adult Gender | -0.12 | 0.04 | -0.20 – -0.04 | **.004** |
| Infant Gender | 0.04 | 0.05 | -0.05 – 0.14 | .362 |
| Adult Gender*Infant Gender | -0.08 | 0.04 | -0.16 – -0.01 | **.033** |
| **Random Effects** | | | | |
| σ^2^ | 0.75 | | | |
| τ_00_ _ID_ | 0.08 | | | |
| τ_00_ _coder_ | 0.00 | | | |
| N _ID_ | 59 | | | |
| N _coder_ | 4 | | | |
| Observations | 900 | | | |
| Marginal R^2^ / Conditional R^2^ | 0.030 / NA | | | |

**Perceived Adult Speaker Gender*Assigned Infant Gender Interaction**

| **Interaction Contrasts** | **Levels** | **Estimate** | **SE** | **DF** | **lower.CL** | **upper.CL** | **t.ratio** | **p.value** |
| --- | --- | --- | --- | --- | --- | --- | --- | --- |
| Adult Female- Adult Male | Infant Females | 0.002 | 0.15 | 221 | -0.34 | 0.34 | 0.01 | .99 |
| Adult Female- Adult Male | Infant Males | 0.41 | 0.12 | 191 | 0.13 | 0.68 | 3.37 | .002 |
| Infant Female- Infant Male | Adult Females | -0.20 | 0.11 | 59.7 | -0.46 | 0.06 | -1.77 | .16 |
| Infant Female- Infant Male | Adult Males | 0.21 | 0.18 | 105.7 | -0.20 | 0.61 | 1.15 | .25 |


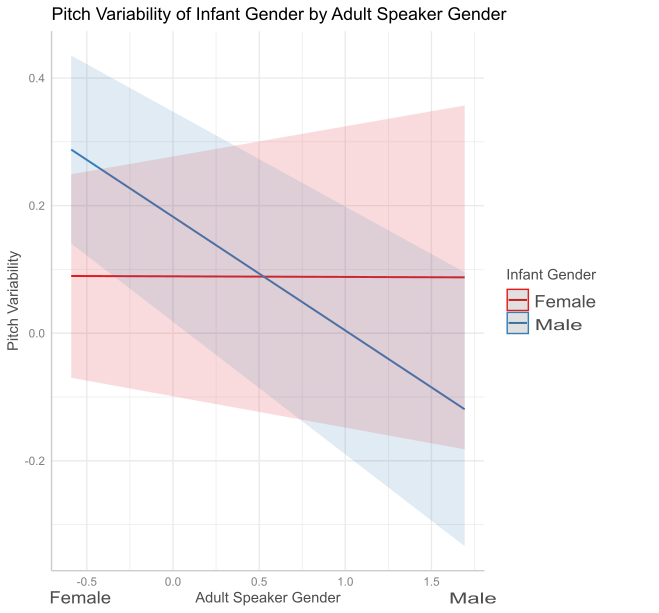

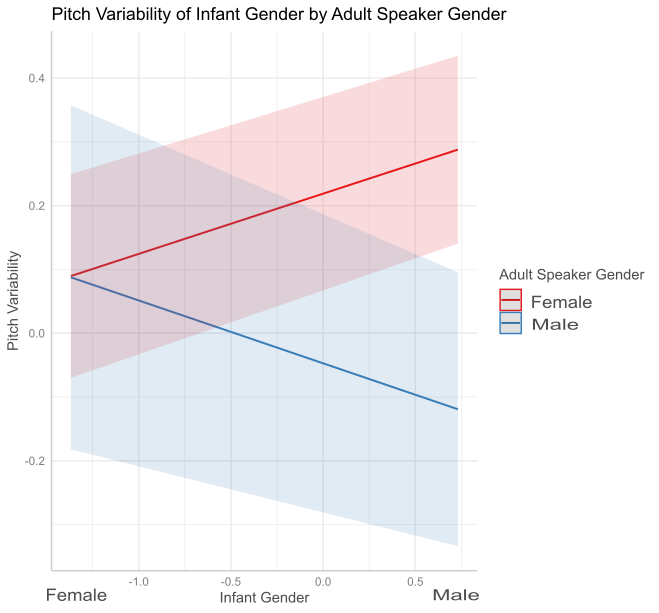


**Conversational Basics**

|  | **Mean Pitch** | | | |  |
| --- | --- | --- | --- | --- | --- |
| *Predictors* | *Estimates* | *SE* | *CI* | *p* | |
| (Intercept) | 0.27 | 0.09 | 0.05 – 0.49 | **.026** | |
| Adult Gender | -0.41 | 0.05 | -0.52 – -0.31 | **<.001** | |
| Infant Gender | 0.04 | 0.06 | -0.09 – 0.17 | .529 | |
| Adult Gender*Infant Gender | 0.08 | 0.05 | -0.01 – 0.18 | .093 | |
| **Random Effects** | | | | | |
| σ^2^ | 0.66 | | | | |
| τ_00_ _ID_ | 0.09 | | | | |
| τ_00_ _coder_ | 0.01 | | | | |
| ICC | 0.13 | | | | |
| N _ID_ | 54 | | | | |
| N _coder_ | 4 | | | | |
| Observations | 359 | | | | |
| Marginal R^2^ / Conditional R^2^ | 0.216 / 0.318 | | | | |

|  | **Pitch Variability** | | | |
| --- | --- | --- | --- | --- |
| *Predictors* | *Estimates* | *SE* | *CI* | *p* |
| (Intercept) | 0.24 | 0.10 | -0.05 – 0.52 | .082 |
| Adult Gender | -0.08 | 0.05 | -0.17 – 0.01 | .080 |
| Infant Gender | 0.06 | 0.05 | -0.04 – 0.17 | .220 |
| Adult Gender*Infant Gender | 0.02 | 0.04 | -0.07 – 0.11 | .651 |
| **Random Effects** | | | | |
| σ^2^ | 0.62 | | | |
| τ_00_ _ID_ | 0.04 | | | |
| τ_00_ _coder_ | 0.03 | | | |
| ICC | 0.09 | | | |
| N _ID_ | 54 | | | |
| N _coder_ | 4 | | | |
| Observations | 359 | | | |
| Marginal R^2^ / Conditional R^2^ | 0.019 / 0.108 | | | |

**Reading**

|  | **Mean Pitch** | | | |  |
| --- | --- | --- | --- | --- | --- |
| *Predictors* | *Estimates* | *SE* | *CI* | *p* | |
| (Intercept) | -0.09 | 0.16 | -0.45 – 0.28 | .603 | |
| Adult Gender | -0.44 | 0.13 | -0.72 – -0.16 | **.003** | |
| Infant Gender | 0.09 | 0.16 | -0.26 – 0.44 | .583 | |
| Adult Gender*Infant Gender | 0.02 | 0.12 | -0.23 – 0.27 | .887 | |
| **Random Effects** | | | | | |
| σ^2^ | 0.74 | | | | |
| τ_00_ _ID_ | 0.30 | | | | |
| τ_00_ _coder_ | 0.00 | | | | |
| ICC | 0.29 | | | | |
| N _ID_ | 14 | | | | |
| N _coder_ | 3 | | | | |
| Observations | 232 | | | | |
| Marginal R^2^ / Conditional R^2^ | 0.161 / 0.402 | | | | |

|  | **Pitch Variability** | | | |
| --- | --- | --- | --- | --- |
| *Predictors* | *Estimates* | *SE* | *CI* | *p* |
| (Intercept) | 0.12 | 0.14 | -0.21 – 0.45 | .421 |
| Adult Gender | -0.11 | 0.12 | -0.36 – 0.14 | .351 |
| Infant Gender | -0.02 | 0.14 | -0.33 – 0.29 | .883 |
| Adult Gender*Infant Gender | 0.01 | 0.11 | -0.21 – 0.23 | .927 |
| **Random Effects** | | | | |
| σ^2^ | 0.61 | | | |
| τ_00_ _ID_ | 0.22 | | | |
| τ_00_ _coder_ | 0.00 | | | |
| N _ID_ | 14 | | | |
| N _coder_ | 3 | | | |
| Observations | 232 | | | |
| Marginal R^2^ / Conditional R^2^ | 0.021 / NA | | | |

**Singing**

|  | **Mean Pitch** | | | |
| --- | --- | --- | --- | --- |
| *Predictors* | *Estimates* | *SE* | *CI* | *p* |
| (Intercept) | 0.13 | 0.12 | -0.17 – 0.44 | 0322 |
| Adult Gender | -0.49 | 0.08 | -0.66 – -0.32 | **< .001** |
| Infant Gender | 0.09 | 0.10 | -0.12 – 0.30 | .383 |
| Adult Gender*Infant Gender | 0.23 | 0.07 | 0.08 – 0.37 | **.002** |
| **Random Effects** | | | | |
| σ^2^ | 0.45 | | | |
| τ_00_ _ID_ | 0.14 | | | |
| τ_00_ _coder_ | 0.01 | | | |
| ICC | 0.25 | | | |
| N _ID_ | 24 | | | |
| N _coder_ | 4 | | | |
| Observations | 224 | | | |
| Marginal R^2^ / Conditional R^2^ | 0.447 / 0.585 | | | |

**Perceived Adult Speaker Gender*Assigned Infant Gender Interaction**

| **Interaction Contrasts** | **Levels** | **Estimate** | **SE** | **DF** | **lower.CL** | **upper.CL** | **t.ratio** | **p.value** |
| --- | --- | --- | --- | --- | --- | --- | --- | --- |
|  |  |  |  |  |  |  |  |  |
| Adult Female- Adult Male | Infant Females | 1.83 | 0.24 | 30.5 | 1.27 | 2.39 | 7.76 | <.001 |
| Adult Female- Adult Male | Infant Males | 0.74 | 0.29 | 38.2 | 0.06 | 1.42 | 2.54 | .02 |
| Infant Female- Infant Male | Adult Females | 0.09 | 0.27 | 19.3 | -0.56 | 0.75 | 0.35 | .73 |
| Infant Female- Infant Male | Adult Males | -0.99 | 0.31 | 28.0 | -1.72 | -0.27 | -3.26 | .006 |


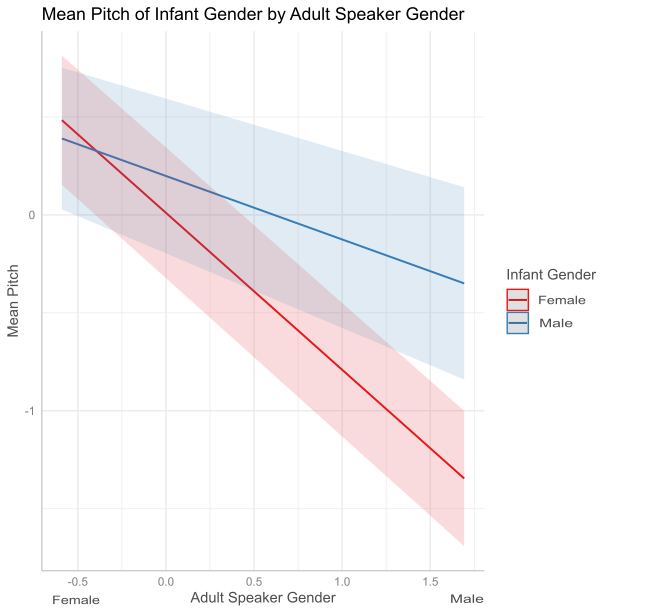

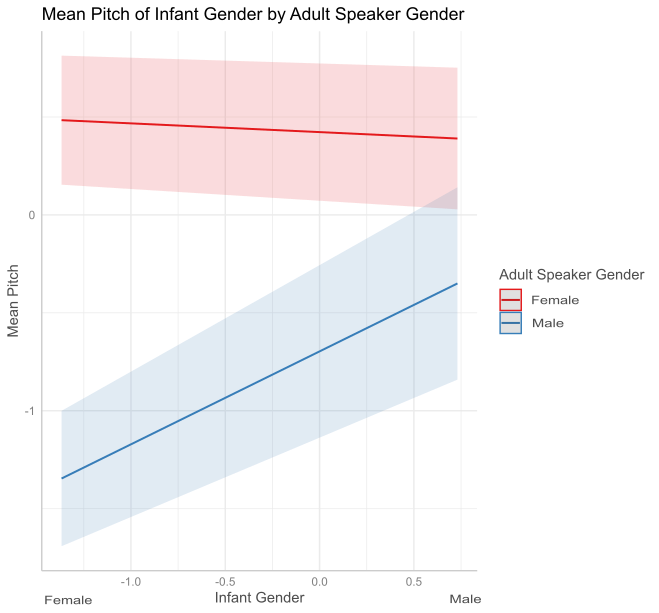


|  | **Pitch Variability** | | | |
| --- | --- | --- | --- | --- |
| *Predictors* | *Estimates* | *SE* | *CI* | *p* |
| (Intercept) | -0.59 | 0.15 | -0.90 – -0.28 | **.001** |
| Adult Gender | -0.35 | 0.12 | -0.60 – -0.10 | **.007** |
| Infant Gender | -0.05 | 0.13 | -0.32 – 0.23 | .727 |
| Adult Gender*Infant Gender | 0.01 | 0.10 | -0.20 – 0.22 | .904 |
| **Random Effects** | | | | |
| σ^2^ | 1.37 | | | |
| τ_00_ _ID_ | 0.18 | | | |
| τ_00_ _coder_ | 0.00 | | | |
| N _ID_ | 24 | | | |
| N _coder_ | 4 | | | |
| Observations | 224 | | | |
| Marginal R^2^ / Conditional R^2^ | 0.089 / NA | | | |

**Question**

|  | | **Mean Pitch** | | | |
| --- | --- | --- | --- | --- | --- |
| *Predictors* | *Estimates* | | *SE* | *CI* | *p* |
| (Intercept) | 0.20 | | 0.07 | 0.06 – 0.34 | **.005** |
| Adult Gender | -0.51 | | 0.04 | -0.59 – -0.42 | **< .001** |
| Infant Gender | 0.06 | | 0.06 | -0.07 – 0.19 | .348 |
| Adult Gender*Infant Gender | 0.05 | | 0.04 | -0.03 – 0.13 | .251 |
| **Random Effects** | | | | | |
| σ^2^ | 0.58 | | | | |
| τ_00_ _ID_ | 0.15 | | | | |
| τ_00_ _coder_ | 0.00 | | | | |
| N _ID_ | 58 | | | | |
| N _coder_ | 4 | | | | |
| Observations | 533 | | | | |
| Marginal R^2^ / Conditional R^2^ | 0.314 / NA | | | | |
|  |  | | | | |

|  | **Pitch Variability** | | | |
| --- | --- | --- | --- | --- |
| *Predictors* | *Estimates* | *SE* | *CI* | *p* |
| (Intercept) | 0.16 | 0.06 | 0.04 – 0.28 | **.011** |
| Adult Gender | -0.07 | 0.05 | -0.16 – 0.02 | .151 |
| Infant Gender | 0.08 | 0.06 | -0.04 – 0.19 | .178 |
| Adult Gender*Infant Gender | -0.03 | 0.04 | -0.12 – 0.06 | .497 |
| **Random Effects** | | | | |
| σ^2^ | 0.74 | | | |
| τ_00_ _ID_ | 0.08 | | | |
| τ_00_ _coder_ | 0.00 | | | |
| N _ID_ | 58 | | | |
| N _coder_ | 4 | | | |
| Observations | 533 | | | |
| Marginal R^2^ / Conditional R^2^ | 0.015 / NA | | | |

**Imperative**

|  | **Mean Pitch** | | | |
| --- | --- | --- | --- | --- |
| *Predictors* | *Estimates* | *SE* | *CI* | *p* |
| (Intercept) | 0.21 | 0.09 | 0.04 – 0.39 | **.019** |
| Adult Gender | -0.45 | 0.06 | -0.57 – -0.33 | **< .001** |
| Infant Gender | 0.01 | 0.08 | -0.16 – 0.18 | .906 |
| Adult Gender*Infant Gender | 0.03 | 0.06 | -0.08 – 0.14 | .591 |
| **Random Effects** | | | | |
| σ^2^ | 0.43 | | | |
| τ_00_ _ID_ | 0.27 | | | |
| τ_00_ _coder_ | 0.00 | | | |
| N _ID_ | 49 | | | |
| N _coder_ | 4 | | | |
| Observations | 349 | | | |
| Marginal R^2^ / Conditional R^2^ | 0.305 / NA | | | |

|  | **Pitch Variability** | | | |
| --- | --- | --- | --- | --- |
| *Predictors* | *Estimates* | *SE* | *CI* | *p* |
| (Intercept) | 0.17 | 0.07 | 0.04 – 0.31 | **.016** |
| Adult Gender | -0.07 | 0.06 | -0.19 – 0.06 | .297 |
| Infant Gender | 0.01 | 0.07 | -0.12 – 0.14 | .887 |
| Adult Gender*Infant Gender | -0.02 | 0.06 | -0.14 – 0.09 | .685 |
| **Random Effects** | | | | |
| σ^2^ | 0.77 | | | |
| τ_00_ _ID_ | 0.09 | | | |
| τ_00_ _coder_ | 0.00 | | | |
| N _ID_ | 49 | | | |
| N _coder_ | 4 | | | |
| Observations | 349 | | | |
| Marginal R^2^ / Conditional R^2^ | 0.006 / NA | | | |

**Comfort**

|  | **Mean Pitch** | | | |
| --- | --- | --- | --- | --- |
| *Predictors* | *Estimates* | *SE* | *CI* | *p* |
| (Intercept) | 0.58 | 0.34 | -0.21 – 1.37 | .130 |
| Adult Gender | -0.57 | 0.27 | -1.19 – 0.05 | .069 |
| Infant Gender | 0.07 | 0.29 | -0.60 – 0.75 | .805 |
| Adult Gender*Infant Gender | 0.34 | 0.23 | -0.19 – 0.86 | .181 |
| **Random Effects** | | | | |
| σ^2^ | 0.63 | | | |
| τ_00_ _ID_ | 0.44 | | | |
| τ_00_ _coder_ | 0.00 | | | |
| N _ID_ | 11 | | | |
| N _coder_ | 4 | | | |
| Observations | 40 | | | |
| Marginal R^2^ / Conditional R^2^ | 0.660 / NA | | | |

|  | **Pitch Variability** | | | |
| --- | --- | --- | --- | --- |
| *Predictors* | *Estimates* | *SE* | *CI* | *p* |
| (Intercept) | 0.69 | 0.44 | -0.36 – 1.75 | .162 |
| Adult Gender | -0.44 | 0.36 | -1.26 – 0.39 | .255 |
| Infant Gender | -0.01 | 0.37 | -0.92 – 0.89 | .969 |
| Adult Gender*Infant Gender | -0.01 | 0.30 | -0.71 – 0.69 | .968 |
| **Random Effects** | | | | |
| σ^2^ | 1.03 | | | |
| τ_00_ _ID_ | 0.74 | | | |
| τ_00_ _coder_ | 0.00 | | | |
| N _ID_ | 11 | | | |
| N _coder_ | 4 | | | |
| Observations | 40 | | | |
| Marginal R^2^ / Conditional R^2^ | 0.188 / NA | | | |

**Alternative Models for Acoustic Analyses**

***Perceived Adult Speaker Gender and Register Analysis Within Specific Pragmatic Contexts: Alternative Model 1***

This model includes all contexts, as well as register and perceived adult gender as predictors, together with three-way interactions between the factors. We decided not to focus on trying to interpret this model because the three-way interactions were not informative and we wished to prioritize the identification of patterns of interaction between register and perceived adult gender within a context, addressing the question of whether there are significant differences across contexts in a separate model focused only on IDS, in order for the results to be more readily interpretable.

Log(mean pitch) ~ register*adult speaker gender*conversational basics + register*adult speaker gender*singing + register*adult speaker gender*inform + register*adult speaker gender*imperative + register*adult speaker gender*question + register*adult speaker gender*reading + register*adult speaker gender*comfort+ register*adult speaker gender*vocal play+ (1 | participant)

|  | **Mean Pitch** | | | |
| --- | --- | --- | --- | --- |
| *Predictors* | *Estimates* | *SE* | *CI* | *p* |
| (Intercept) | 0.04 | 0.05 | -0.05 – 0.13 | .402 |
| Adult Gender | -0.43 | 0.02 | -0.48 – -0.39 | **< .001** |
| Conversational Basics | 0.04 | 0.01 | 0.01 – 0.07 | **.003** |
| Register | 0.15 | 0.03 | 0.10 – 0.21 | **< .001** |
| Sing | 0.02 | 0.06 | -0.10 – 0.14 | .710 |
| Inform | -0.03 | 0.02 | -0.06 – 0.01 | .200 |
| Read | -0.02 | 0.02 | -0.06 – 0.02 | .263 |
| Imperative | 0.04 | 0.02 | 0.00 – 0.08 | **.031** |
| Question | -0.00 | 0.02 | -0.04 – 0.03 | .848 |
| Comfort | 0.04 | 0.03 | -0.01 – 0.09 | .130 |
| Vocal Play | 0.00 | 0.01 | -0.03 – 0.03 | .847 |
| Adult Gender*Conversational Basics | 0.04 | 0.01 | 0.01 – 0.06 | **.014** |
| Adult Gender*Register | -0.04 | 0.02 | -0.09 – 0.00 | .078 |
| Conversational Basics*Register | -0.01 | 0.01 | -0.04 – 0.01 | .359 |
| Adult Gender*Sing | -0.11 | 0.05 | -0.21 – -0.02 | **.021** |
| Register*Sing | -0.04 | 0.08 | -0.19 – 0.11 | .573 |
| Adult Gender*Inform | -0.03 | 0.02 | -0.06 – 0.01 | .188 |
| Register*Inform | 0.01 | 0.02 | -0.03 – 0.05 | .524 |
| Adult Gender*Read | -0.03 | 0.02 | -0.07 – 0.00 | .077 |
| Adult Gender*Imperative | 0.01 | 0.02 | -0.04 – 0.05 | .804 |
| Register*Imperative | -0.04 | 0.02 | -0.09 – 0.00 | .058 |
| Adult Gender*Question | -0.04 | 0.02 | -0.08 – -0.01 | **.008** |
| Register*Question | -0.00 | 0.02 | -0.03 – 0.03 | .929 |
| Register*Comfort | -0.03 | 0.03 | -0.10 – 0.03 | .293 |
| Adult Gender*Comfort | -0.04 | 0.01 | -0.06 – -0.01 | **.009** |
| Adult Gender*Vocal Play | 0.02 | 0.01 | -0.01 – 0.05 | .140 |
| Adult Gender*Conversational Basics*Register | -0.02 | 0.01 | -0.05 – 0.00 | .107 |
| Adult Gender*Register*Sing | 0.08 | 0.06 | -0.03 – 0.20 | .163 |
| Adult Gender*Register*Inform | 0.01 | 0.02 | -0.03 – 0.05 | .782 |
| Adult Gender*Register*Imperative | -0.00 | 0.03 | -0.05 – 0.05 | .990 |
| Adult Gender*Register*Question | 0.03 | 0.02 | -0.00 – 0.07 | .060 |
| **Random Effects** | | | | |
| σ^2^ | 0.67 | | | |
| τ_00_ _ID_ | 0.09 | | | |
| τ_00_ _coder_ | 0.00 | | | |
| N _ID_ | 60 | | | |
| N _coder_ | 4 | | | |
| Observations | 3607 | | | |
| Marginal R^2^ / Conditional R^2^ | 0.251 / NA | | | |

Log(pitch standard deviation) ~ register*adult speaker gender*conversational basics + register*adult speaker gender*singing + register*adult speaker gender*inform + register*adult speaker gender*imperative + register*adult speaker gender*question + register*adult speaker gender*reading + register*adult speaker gender*comfort+ register*adult speaker gender*vocal play+ (1 | participant)

|  | **Pitch Variability** | | | |
| --- | --- | --- | --- | --- |
| *Predictors* | *Estimates* | *SE* | *CI* | *p* |
| (Intercept) | 0.05 | 0.05 | -0.04 – 0.14 | .276 |
| Adult Gender | -0.07 | 0.03 | -0.12 – -0.02 | **.009** |
| Conversational Basics | 0.07 | 0.02 | 0.04 – 0.10 | **< .001** |
| Register | 0.07 | 0.03 | 0.01 – 0.13 | **.025** |
| Sing | -0.06 | 0.07 | -0.20 – 0.08 | .406 |
| Inform | -0.01 | 0.02 | -0.05 – 0.04 | .783 |
| Read | 0.02 | 0.02 | -0.02 – 0.07 | .271 |
| Imperative | 0.03 | 0.02 | -0.01 – 0.08 | .129 |
| Question | 0.01 | 0.02 | -0.03 – 0.04 | .735 |
| Comfort | 0.10 | 0.03 | 0.04 – 0.16 | **.001** |
| Vocal Play | 0.00 | 0.02 | -0.03 – 0.04 | .869 |
| Adult Gender*Conversational Basics | 0.01 | 0.02 | -0.02 – 0.05 | .433 |
| Adult Gender*Register | -0.02 | 0.03 | -0.07 – 0.03 | .412 |
| Conversational Basics*Register | -0.03 | 0.02 | -0.06 – 0.00 | .087 |
| Adult Gender*Sing | -0.07 | 0.06 | -0.18 – 0.04 | .243 |
| Register*Sing | -0.16 | 0.09 | -0.34 – 0.01 | .066 |
| Adult Gender*Inform | 0.04 | 0.02 | -0.01 – 0.08 | .108 |
| Register*Inform | -0.01 | 0.02 | -0.06 – 0.04 | .685 |
| Adult Gender*Read | -0.02 | 0.02 | -0.06 – 0.02 | .407 |
| Adult Gender*Imperative | 0.06 | 0.02 | 0.01 – 0.11 | **.018** |
| Register*Imperative | -0.04 | 0.03 | -0.09 – 0.01 | .121 |
| Adult Gender*Question | -0.00 | 0.02 | -0.04 – 0.03 | .834 |
| Register*Question | -0.02 | 0.02 | -0.06 – 0.02 | .290 |
| Register*Comfort | -0.06 | 0.04 | -0.14 – 0.01 | .097 |
| Adult Gender*Comfort | -0.01 | 0.02 | -0.04 – 0.02 | .674 |
| Adult Gender*Vocal Play | -0.00 | 0.02 | -0.03 – 0.03 | .947 |
| Adult Gender*Conversational Basics*Register | -0.00 | 0.02 | -0.03 – 0.03 | .922 |
| Adult Gender*Register*Sing | 0.02 | 0.07 | -0.12 – 0.16 | .768 |
| Adult Gender*Register*Inform | -0.01 | 0.02 | -0.06 – 0.03 | .574 |
| Adult Gender*Register*Imperative | -0.06 | 0.03 | -0.11 – -0.00 | **.048** |
| Adult Gender*Register*Question | 0.02 | 0.02 | -0.02 – 0.06 | .363 |
| **Random Effects** | | | | |
| σ^2^ | 0.89 | | | |
| τ_00_ _ID_ | 0.05 | | | |
| τ_00_ _coder_ | 0.00 | | | |
| ICC | 0.05 | | | |
| N _ID_ | 60 | | | |
| N _coder_ | 4 | | | |
| Observations | 3607 | | | |
| Marginal R^2^ / Conditional R^2^ | 0.066 / 0.116 | | | |

***Perceived Adult Speaker Gender and Register Analysis Within Specific Pragmatic Contexts: Alternative Model 2***

This model includes the four contexts with at least 20 instances in both IDS and ADS, as well as register and perceived adult gender as predictors, together with three-way interactions between the factors. As with Alternative Model 1, we decided not to focus on trying to interpret this model because the three-way interactions were minimally informative and we wished to prioritize the identification of patterns of interaction between register and perceived adult gender within a context, addressing the question of whether there are significant differences across contexts in a separate model focused only on IDS, in order for the results to be more readily interpretable.

Log(mean pitch) ~ Adult Speaker Gender*Register*Conversational Basics + Adult Speaker Gender* Register*Inform + Adult Speaker Gender*Register*Imperative + Adult Speaker Gender*Register*Question + (1 | ID) + (1 | coder)

|  | **Mean Pitch** | | | |
| --- | --- | --- | --- | --- |
| *Predictors* | *Estimates* | *SE* | *CI* | *p* |
| (Intercept) | 0.03 | 0.05 | -0.06 – 0.12 | .505 |
| Adult Gender | -0.41 | 0.02 | -0.45 – -0.38 | **< .001** |
| Register | 0.16 | 0.02 | 0.12 – 0.19 | **< .001** |
| Conversational Basics | 0.05 | 0.01 | 0.02 – 0.08 | **.001** |
| Inform | -0.02 | 0.02 | -0.06 – 0.01 | .256 |
| Imperative | 0.05 | 0.02 | 0.01 – 0.08 | **.016** |
| Question | 0.00 | 0.02 | -0.03 – 0.03 | .873 |
| Adult Gender*Register | -0.07 | 0.02 | -0.11 – -0.04 | **< .001** |
| Adult Gender*Conversational Basics | 0.05 | 0.01 | 0.02 – 0.08 | **.001** |
| Register*Conversational Basics | -0.01 | 0.01 | -0.03 – 0.02 | .622 |
| Adult Gender*Inform | -0.00 | 0.02 | -0.04 – 0.03 | .849 |
| Register*Inform | 0.02 | 0.02 | -0.01 – 0.06 | .205 |
| Adult Gender*Imperative | 0.02 | 0.02 | -0.02 – 0.06 | .398 |
| Register*Imperative | -0.04 | 0.02 | -0.08 – 0.01 | .092 |
| Adult Gender*Question | -0.03 | 0.02 | -0.06 – 0.00 | .081 |
| Register*Question | 0.01 | 0.02 | -0.03 – 0.04 | .715 |
| Adult Gender*Register*Conversational Basics | -0.02 | 0.01 | -0.04 – 0.01 | .277 |
| Adult Gender*Register*Inform | 0.02 | 0.02 | -0.02 – 0.06 | .354 |
| Adult Gender*Register*Imperative | 0.01 | 0.02 | -0.04 – 0.06 | .750 |
| Adult Gender*Register*Question | 0.04 | 0.02 | 0.01 – 0.08 | **.007** |
| **Random Effects** | | | | |
| σ^2^ | 0.67 | | | |
| τ_00_ _ID_ | 0.10 | | | |
| τ_00_ _coder_ | 0.00 | | | |
| ICC | 0.13 | | | |
| N _ID_ | 60 | | | |
| N _coder_ | 4 | | | |
| Observations | 3607 | | | |
| Marginal R^2^ / Conditional R^2^ | 0.218 / 0.322 | | | |

Log(pitch standard deviation) ~ Adult Speaker Gender*Register*Conversational Basics + Adult Speaker Gender* Register*Inform + Adult Speaker Gender*Register*Imperative + Adult Speaker Gender*Register*Question + (1 | ID) + (1 | coder)

|  | **Pitch Variability** | | | |
| --- | --- | --- | --- | --- |
| *Predictors* | *Estimates* | *SE* | *CI* | *p* |
| (Intercept) | 0.02 | 0.04 | -0.07 – 0.11 | .674 |
| Adult Gender | -0.07 | 0.02 | -0.11 – -0.03 | **.002** |
| Register | 0.09 | 0.02 | 0.05 – 0.13 | **< .001** |
| Conversational Basics | 0.09 | 0.02 | 0.06 – 0.12 | **< .001** |
| Inform | 0.03 | 0.02 | -0.02 – 0.07 | .219 |
| Imperative | 0.06 | 0.02 | 0.01 – 0.10 | **.013** |
| Question | 0.04 | 0.02 | 0.00 – 0.07 | **.047** |
| Adult Gender*Register | -0.05 | 0.02 | -0.09 – -0.01 | **.021** |
| Adult Gender*Conversational Basics | 0.02 | 0.02 | -0.01 – 0.06 | .152 |
| Register*Conversational Basics | -0.01 | 0.02 | -0.04 – 0.02 | .591 |
| Adult Gender*Inform | 0.05 | 0.02 | 0.01 – 0.09 | **.021** |
| Register*Inform | 0.02 | 0.02 | -0.02 – 0.07 | .274 |
| Adult Gender*Imperative | 0.07 | 0.02 | 0.02 – 0.12 | **.004** |
| Register*Imperative | -0.02 | 0.03 | -0.07 – 0.03 | .414 |
| Adult Gender*Question | 0.01 | 0.02 | -0.03 – 0.04 | .712 |
| Register*Question | 0.00 | 0.02 | -0.03 – 0.04 | .801 |
| Adult Gender*Register*Conversational Basics | 0.01 | 0.02 | -0.03 – 0.04 | .733 |
| Adult Gender*Register*Inform | -0.01 | 0.02 | -0.06 – 0.03 | .599 |
| Adult Gender*Register*Imperative | -0.05 | 0.03 | -0.10 – 0.01 | .097 |
| Adult Gender*Register*Question | 0.03 | 0.02 | -0.01 – 0.06 | .192 |
| **Random Effects** | | | | |
| σ^2^ | 0.91 | | | |
| τ_00_ _ID_ | 0.06 | | | |
| τ_00_ _coder_ | 0.00 | | | |
| ICC | 0.06 | | | |
| N _ID_ | 60 | | | |
| N _coder_ | 4 | | | |
| Observations | 3607 | | | |
| Marginal R^2^ / Conditional R^2^ | 0.032 / 0.094 | | | |

***IDS and Assigned Infant Gender: Alternative Model***

This model includes perceived adult gender, assigned child (infant) gender, and several contexts (included if they met our minimum number of samples criterion) in one model. As with the Alternative Models for the register*gender analyses, we decided not to focus on trying to interpret this model because the three-way interactions were minimally informative and we wished to prioritize the identification of any infant gender effects within any context. This was more important to us than knowing if such infant gender effects were significantly different across contexts.

Log(Mean Pitch) ~ Adult Gender*Child Gender*Conversational Basics + Adult Gender*Child Gender*Inform + Adult Gender*Child Gender*Question + Adult Gender*Child Gender*Imperative + Adult Gender*Child Gender*Read + Adult Gender*Child Gender*Comfort + (1|ID)+ (1|coder)

|  | **Mean Pitch** | | | |
| --- | --- | --- | --- | --- |
| *Predictors* | *Estimates* | *SE* | *CI* | *p* |
| (Intercept) | 0.20 | 0.07 | 0.06 – 0.33 | **.004** |
| Adult Gender | -0.47 | 0.03 | -0.52 – -0.42 | **< .001** |
| Child Gender | 0.03 | 0.05 | -0.06 – 0.12 | .534 |
| Conversational Basics | 0.04 | 0.02 | -0.00 – 0.07 | .077 |
| Inform | -0.01 | 0.02 | -0.05 – 0.03 | .594 |
| Question | 0.00 | 0.02 | -0.03 – 0.04 | .813 |
| Imperative | 0.01 | 0.02 | -0.02 – 0.05 | .390 |
| Read | -0.03 | 0.02 | -0.07 – 0.00 | .079 |
| Comfort | 0.02 | 0.02 | -0.02 – 0.07 | .260 |
| Adult Gender*Child Gender | 0.07 | 0.03 | 0.02 – 0.12 | **.005** |
| Adult Gender*Conversational Basics | 0.02 | 0.02 | -0.02 – 0.06 | .238 |
| Child Gender*Conversational Basics | 0.00 | 0.02 | -0.04 – 0.04 | .918 |
| Adult Gender*Inform | -0.02 | 0.02 | -0.06 – 0.02 | .404 |
| Child Gender*Inform | 0.02 | 0.02 | -0.02 – 0.06 | .299 |
| Adult Gender*Question | -0.01 | 0.02 | -0.05 – 0.03 | .521 |
| Child Gender*Question | 0.03 | 0.02 | -0.01 – 0.06 | .133 |
| Adult Gender*Imperative | 0.02 | 0.02 | -0.02 – 0.05 | .387 |
| Child Gender*Imperative | -0.00 | 0.02 | -0.04 – 0.03 | .827 |
| Adult Gender*Read | -0.02 | 0.02 | -0.06 – 0.01 | .204 |
| Child Gender*Read | 0.03 | 0.02 | -0.01 – 0.07 | .134 |
| Adult Gender*Comfort | -0.00 | 0.02 | -0.04 – 0.04 | .874 |
| Child Gender*Comfort | 0.01 | 0.02 | -0.03 – 0.04 | .673 |
| Adult Gender*Child Gender*Conversational Basics | -0.00 | 0.02 | -0.04 – 0.03 | .825 |
| Adult Gender*Child Gender*Inform | -0.01 | 0.02 | -0.05 – 0.03 | .538 |
| Adult Gender*Child Gender*Question | -0.02 | 0.02 | -0.05 – 0.02 | .336 |
| Adult Gender*Child Gender*Imperative | -0.02 | 0.02 | -0.05 – 0.02 | .304 |
| Adult Gender*Child Gender*Read | -0.02 | 0.02 | -0.05 – 0.02 | .419 |
| Adult Gender*Child Gender*Comfort | 0.03 | 0.02 | -0.00 – 0.06 | .071 |
| **Random Effects** | | | | |
| σ^2^ | 0.68 | | | |
| τ_00_ _ID_ | 0.11 | | | |
| τ_00_ _coder_ | 0.01 | | | |
| ICC | 0.14 | | | |
| N _ID_ | 60 | | | |
| N _coder_ | 4 | | | |
| Observations | 2210 | | | |
| Marginal R^2^ / Conditional R^2^ | 0.244 / 0.353 | | | |

Log(Standard Deviation of Pitch) ~ Adult Gender*Child Gender*Conversational Basics + Adult Gender*Child Gender*Inform + Adult Gender*Child Gender*Question + Adult Gender*Child Gender*Imperative + Adult Gender*Child Gender*Read + Adult Gender*Child Gender*Comfort + (1|ID)+ (1|coder)

|  | **Pitch Variability** | | | |
| --- | --- | --- | --- | --- |
| *Predictors* | *Estimates* | *SE* | *CI* | *p* |
| (Intercept) | 0.10 | 0.04 | 0.02 – 0.19 | **.020** |
| Adult Gender | -0.11 | 0.03 | -0.16 – -0.05 | **<.001** |
| Child Gender | 0.04 | 0.04 | -0.05 – 0.12 | .384 |
| Conversational Basics | 0.09 | 0.02 | 0.04 – 0.13 | **<.001** |
| Inform | 0.06 | 0.02 | 0.01 – 0.11 | **.015** |
| Question | 0.07 | 0.02 | 0.03 – 0.11 | **.001** |
| Imperative | 0.05 | 0.02 | 0.01 – 0.09 | **.010** |
| Read | 0.05 | 0.02 | 0.01 – 0.09 | **.014** |
| Comfort | 0.08 | 0.02 | 0.04 – 0.13 | **.001** |
| Adult Gender*Child Gender | -0.00 | 0.03 | -0.06 – 0.05 | .943 |
| Adult Gender*Conversational Basics | 0.02 | 0.02 | -0.02 – 0.07 | .291 |
| Child Gender*Conversational Basics | -0.00 | 0.02 | -0.05 – 0.04 | .896 |
| Adult Gender*Inform | 0.03 | 0.02 | -0.02 – 0.07 | .304 |
| Child Gender*Inform | -0.00 | 0.02 | -0.05 – 0.04 | .839 |
| Adult Gender*Question | 0.02 | 0.02 | -0.02 – 0.06 | .383 |
| Child Gender*Question | 0.02 | 0.02 | -0.02 – 0.06 | .346 |
| Adult Gender*Imperative | 0.03 | 0.02 | -0.01 – 0.07 | .157 |
| Child Gender*Imperative | -0.01 | 0.02 | -0.04 – 0.03 | .751 |
| Adult Gender*Read | -0.01 | 0.02 | -0.05 – 0.03 | .739 |
| Child Gender*Read | 0.00 | 0.02 | -0.04 – 0.05 | .916 |
| Adult Gender*Comfort | -0.04 | 0.02 | -0.08 – 0.00 | .069 |
| Child Gender*Comfort | 0.03 | 0.02 | -0.01 – 0.07 | .089 |
| Adult Gender*Child Gender*Conversational Basics | 0.01 | 0.02 | -0.03 – 0.05 | .514 |
| Adult Gender*Child Gender*Inform | -0.05 | 0.02 | -0.09 – -0.00 | **.050** |
| Adult Gender*Child Gender*Question | -0.03 | 0.02 | -0.07 – 0.01 | .180 |
| Adult Gender*Child Gender*Imperative | -0.02 | 0.02 | -0.05 – 0.02 | .424 |
| Adult Gender*Child Gender*Read | -0.00 | 0.02 | -0.05 – 0.04 | .830 |
| Adult Gender*Child Gender*Comfort | -0.01 | 0.02 | -0.05 – 0.02 | .399 |
| **Random Effects** | | | | |
| σ^2^ | 0.87 | | | |
| τ_00_ _ID_ | 0.07 | | | |
| τ_00_ _coder_ | 0.00 | | | |
| N _ID_ | 60 | | | |
| N _coder_ | 4 | | | |
| Observations | 2210 | | | |
| Marginal R^2^ / Conditional R^2^ | 0.046 / NA | | | |
