# Supplementary material for: Pitch characteristics of real-world infant-directed speech vary with pragmatic context, perceived adult gender, and infant gender
Source: PLoS One. 2025 Jun 25;20(6):e0326569. doi: 10.1371/journal.pone.0326569 (PMC12192138; doi:10.1371/journal.pone.0326569)
Supplement: Attachment — Submitted filename: Response to Reviewers.docx [file pone.0326569.s004.docx]

Dear Editors,

Thank you for offering us the opportunity to revise the manuscript. We are grateful to the Editor and all the reviewers for their helpful comments. Below are responses to the comments and suggestions on the previous revision.

**Reviewer #3 Comments:**

1. The authors note that adult speaker gender was removed from the Bayesian model to achieve convergence—an understandable decision given the data limitations and model complexity. However, since adult gender was central to the study’s original hypotheses and included in the preregistration, it would be helpful to explicitly acknowledge this as a deviation from the preregistered analysis and briefly reflect on its implications for interpreting the results. In addition, I recommend that the authors clearly state that the adoption of the Bayesian approach also constitutes a deviation from the preregistration (Variables and Analyses 1).

Response: Thank you for this suggestion, and we agree that transparency is needed. We have added a statement in the “Preregistered Planned Analyses and Pilot Analyses” section to highlight the adoption of the Bayesian approach and that this approach differs from the preregistration. Additionally, we added a short paragraph in the discussion section on the implications of excluding speaker gender from the analysis.

2. Reproducing the Bayesian analysis requires substantial computational time and resources (taking 11 hours for the Bayesian model to run on my end!). This could pose a barrier to reproducibility for researchers without access to high-performance computing setups. I suggest the authors consider caching model outputs (e.g., using saveRDS() or storing fitted model objects) and including them in the repository. This would facilitate transparency and reproducibility without requiring each user to rerun time-intensive sampling.

Response: Thank you so much for taking the time to run our analyses and for this helpful suggestion. We have taken the suggestion to save the model object using saveRDS() and the file is now included in the GitHub repository associated with the manuscript. We also updated the results output text file on GitHub and have updated the numbers in Table 2 and the reported ESSs to match this most recent run of the model.

3. p. 16: max_treedepth is 15 within the code but reported as 12 in the manuscript
   Scaling and centering of binary predictors such as adult speaker gender, child gender, and register are performed using the scale() function. I am wondering whether effect coding (e.g., −0.5 / 0.5) would typically offer a more interpretable and robust approach in this context?

Response: Thank you for catching our error in reporting the value of max_treedepth! The correction has been made in the manuscript. We also appreciate the suggestion to consider effect coding. While we did not make this change to the effect coding for this manuscript, as it would have been a fairly major revision. However, we will consider taking an effect coding approach in future studies.
